# The Impact of Health Resort Treatment on the Nonenzymatic Endogenous Antioxidant System

**DOI:** 10.1155/2020/8423105

**Published:** 2020-01-31

**Authors:** Jadwiga Kuciel-Lewandowska, Michał Kasperczak, Bożena Bogut, Roman Heider, Wojciech T. Laber, Wojciech Laber, Małgorzata Paprocka-Borowicz

**Affiliations:** Department of Physiotherapy, Medical University of Wrocław, Poland

## Abstract

*Introduction*. Oxygen, reacting with organic compounds in living organisms, oxidizes them without being completely reduced due to numerous exogenous as well as endogenous factors. As a consequence, free radicals or reactive oxygen species are formed. Health resort-based balneophysiotherapy is a comprehensive therapeutic intervention that triggers positive therapeutic effects within the entire system. *Material and Methods*. The objective of the study was to assess the impact of health resort-based balneophysiotherapy on the levels of nonenzymatic endogenous antioxidants in patients with degenerative motor organ diseases, as well as to determine potential correlation of these changes with free radical-mediated processes. Observation was carried out in patients undergoing health resort therapy as part of 21-day stay periods. The study population consisted of *n* = 110 patients with articular and spinal pains due to degenerative diseases or discopathies.

**Results:**

Reduced bilirubin and albumin levels as well as increased uric acid levels were observed in the study group following the health resort treatment.

**Conclusions:**

Bilirubin and albumin levels were reduced while uric acid levels were increased as the result of health resort therapy in patients with degenerative motor organ diseases. The observed changes in the levels of nonenzymatic endogenous antioxidants depend on free radical-mediated systemic transformations. The trial is registered with NCT03405350.

## 1. Introduction

The main source of reactive oxygen species (ROS) consists in cellular respiration processes catalyzed by various enzymes. The effects of free radicals are counterbalanced by antioxidants, i.e., compounds which, albeit being present at low concentrations, markedly inhibit oxidation of specific molecules [1]. Reactive oxygen species include singlet oxygen (^1^O_2_), superoxide anion radical (O_2_^·−^), hydrogen peroxide (H_2_O_2_), and hydroxide radical (^·^OH). In a healthy system, the levels of free radicals are strictly controlled by the maintenance of equilibrium between formation and elimination of reactive oxygen species. Disturbance in this equilibrium is referred to as oxidative stress. Oxidative stress occurs when the level of antioxidants, i.e., substances that fight free radicals, is decreased or when generation of reactive oxygen species intensifies for various reasons [2]. As long as equilibrium between the formation and the elimination of reactive oxygen species is maintained, they remain harmless for the system. A disturbance in this equilibrium leads to manifestations of toxic effects of ROS, e.g., in the motor organ inflammatory diseases. A cascade of enzymatic reactions is triggered that leads, e.g., to depolymerization of hyaluronic acid resulting in the loss of tissue elasticity, degradation of proteoglycans and collagen, oxidation of proteins, or inhibition of chondrocyte proliferation. Free radicals are also involved in the development of other diseases such as atherosclerosis, neurodegenerative diseases such as Alzheimer's or Parkinson's disease, inflammations, allergies, cancers, diabetes, or macular degeneration [3]. The human system is equipped with several mechanisms that regulate the production of free radicals or limit/repair their effects. The antioxidation system consists of several components (antioxidants). One of the possible activities supporting the production of antioxidants is balneophysiotherapy treatments. Balneophysiotherapy includes balneology, physiotherapy, and kinesitherapy treatments. Health resort-based balneophysiotherapy is a comprehensive therapeutic intervention that triggers positive therapeutic effects within the entire system.

Antioxidants are substances which, while being present at low quantities relative to the substrate undergoing oxidation, significantly restrict the oxidation process. Upon systemic equilibrium, free radicals are eliminated or neutralized by different means, such as a decrease in oxygen levels, transformation of radicals into nonradical substances such as alcohols, or binding of iron and copper to inhibit initiation of ^·^OH radical formation. The antioxidation defense system consists of

(1) endogenous antioxidants produced within the system:
  - enzymatic (antioxidative enzymes): superoxide dismutase (SOD), glutathione peroxidase (GSH-Px), and catalase; the main task of these enzymes is to suppress the reactions between free radicals and biological agents contained within cells by metabolizing these radicals into less toxic products
  - nonenzymatic albumin, bilirubin, glutathione, uric acid, ceruloplasmin, transferrin, and coenzyme Q10; the compounds differ in their target molecules [4]
(2) exogenous antioxidants delivered from the outside: vitamins C, A, and E, carotenoids, xanthophylls, and polyphenols. The compounds directly enter free radical reactions and impact the transmission of cell signals and the activity of enzymes and genes involved in cell death and DNA repair processes [5, 6]

Another classification of antioxidants highlights their triple line of defense. The first line of defense consists of antioxidative enzymes; the second line consists of proteins that bind elemental ions such as albumin, ceruloplasmin, or transferrin. The defense mechanism consists in the suppression of free radical formation as well as in the suppression of their reactions with biologically active substances. The third line of defense consists in free radical scavengers. Hydrophilic antioxidants active within the aqueous environment include vitamin C, uric acid, and glutathione, while antioxidants active within lipophilic conditions include carotenoids and vitamin E [7, 8].

The efficacy of the antioxidative system and thus the oxidative stress level may be assessed by either of three different methods:

1. Direct assessment of enzymatic and nonenzymatic antioxidants in blood plasma. Subject to the assessment are the levels of individual antioxidative enzymes and the levels of other antioxidants. Tests are carried out using laboratory diagnostic assay kits that encompass a number of methods, mostly colorimetric in nature, for application in various automated instruments
2. Assessment of total antioxidant status (TAS) of plasma. Subject to the assessment is the integral antioxidative system encompassing all biological agents showing oxidation-preventing activity
3. Determination of oxidative stress biomarkers defined as biomolecules altered by free radicals [9, 10]

It turned out that many compounds hitherto considered to play no particular positive role within the system are in fact excellent antioxidants. They are the end products of certain metabolic pathways, including uric acid (the end product of purin transformations) or bilirubin (the end product of heme transformations).

Uric acid reduces the accumulation of free radicals, stabilizes mitochondrial function, and impacts the calcium ion balance by regulating the permeability of cell membranes; it is also capable of scavenging hydroxyl radicals [11–13] by being a strong reductor (electron donor) and a strong antioxidant [14]. Uric acid is capable of scavenging various reactive oxygen species. Probably, it is capable of forming stable complexes with iron or copper ions, thus inhibiting free radical-mediated reactions such as Fenton reaction or Haber-Weiss reaction [15–17]. Uric acid is not always considered to be an antioxidant since hyperuricemia may be a risk factor of cardiovascular diseases. Some data are suggestive of a relationship between elevated uric acid levels and diabetes, arterial hypertension, and ischemic heart disease [18] while others suggest no relationship between hyperuricemia and atherosclerosis [19].

Bilirubin is primarily a marker of the hepatic function. In high levels, it has toxic properties. Moderate bilirubin levels were ascribed with some beneficial effects including anti-inflammatory, antiatherosclerotic, and antiplatelet effects [20–23]. In biological studies, bilirubin was found to exert strong antioxidative effects [24, 25]. Strong antioxidative potential of bilirubin was observed in relation to superoxide radicals [26]. Bilirubin prevents peroxidation of linolenic acid and deactivates singlet oxygen while being somewhat less efficient in deactivating hypochlorites.

Proteins, including albumin, play an important role in antioxidative protection. Albumin is a multifunctional antioxidative protein which binds redox metals (Fe II and Cu II) and may potentially act as a scavenger and reactant for hydroxyl radicals. Albumin may bind numerous compounds, such as fatty acids which may be formed in free radical-mediated processes, or heme-bound iron. In addition, hydroxide radicals react directly with albumin molecules resulting in molecular damage immediately repaired by production of another albumin molecule. As the main constituent of plasma, albumin is responsible for protection of morphotic blood components [27–31]. The objective of the study was to assess the impact of health resort-based balneophysiotherapy on the levels of nonenzymatic endogenous antioxidants such as bilirubin, albumin, and uric acid, in patients with degenerative motor organ diseases, as well as to determine potential correlation of these changes with free radical-mediated processes.

## 2. Material and Methods

The study was carried out in the Przerzeczyn-Zdroj health resort. Observation was carried out in patients undergoing health resort therapy as part of 21-day stay periods. Blood samples were collected from patients via a venous puncture before and after 21 days of resort treatment. After collection, fresh blood was placed in EDTA-coated tubes and transferred for analysis at +6°C.

The study population consisted of *n* = 110 patients, including 85 males and 25 females with articular and spinal pain due to degeneration of discopathy. The age of the patients ranged from 32 to 67 years, with the mean age of 53.5 years. The selection criteria included degenerative disease of joints and/or spine, absence of contraindications for comprehensive health resort treatment, or lack of metabolic disorders. Exclusion criteria consisted of the presence of disorders considered to be contraindications for the treatment (as per the standard list of indications and contraindications for health resort treatment) or the presence of metabolic disorders. Patients above the age of 80 were not included in the study. Most patients were on standard diet or a light diet with predominance of water-boiled, low-fat meals. Both diets were normocalorific.

The main therapeutic agent used in the treatment consisted of radon- and sulfur-containing thermal water. Procedures included radon- and sulfur-containing water whole-body immersion baths at the temperature of 37°C. The baths were taken by subjects for 15 minutes every second day. A total of 15 radon procedures were delivered during the resort stay period. In addition, the treatment consisted of peat treatment, dry therapeutic massage, kinesitherapy, and physical therapy. Patients were prescribed with a series of 10 procedures of particular types depending on their complaints. An example set of treatment procedures included radon- and sulfur-containing water baths, peat compresses, group and individual gymnastics, laser biostimulation, and interference currents.

Comprehensive resort treatment in the study group patients consisted of the following recommendations:

- Radon- and sulfur-containing water baths with whole body or partial upper and/or lower limb immersion at the temperature of 37-38°C and with the duration of 20 minutes
- Partial peat compresses at the temperature of 40-42°C and with the duration of 20 minutes
- Therapeutic gymnastics in a therapeutically neutral water pool
- Individual apparatus-based and group gymnastics; exercises were customized to each patient with consideration to their individual fitness; the mean duration of kinesitherapy was 30-45 minutes
- Outdoor treatment—walks, outdoor motion classes
- Dry massage—depending on the needs, massage was delivered to the cervical (CC), thoracic (TH), or lumbar spine (LS); duration: 20 minutes
- Laser therapy—parameters: sweeping technique, continuous operation, wavelength 808 nm, 12.0 J power, 400 mV, duration 30 s
- Low-frequency magnetic field: duration 20 min., square pulse, 5 mT induction, frequency 20-50 Hz
- Ultrasound therapy—parameters: 800 kHz/6 cm^2^ probe, pulse wave consisting of ultrasound pulses of 2 ms at 9 ms intervals, dose range 0.5-0.6 W/cm^2^, duration 6 minutes
- Cryotherapy—ventilation, duration 2-3 minutes, temperature from −80°C to −110°C
- Electrotherapy: Bernard's biodynamic currents—parameters: DF1 CP4 LP4, Nemec's interferential currents (frequency range 0-100 Hz); transcutaneous electrical nerve stimulation (TENS)—square-wave pulse current, pulse width 0.2 ms, frequency 40 Hz, intensity regulated within the 0-100 mA range
- Light therapy: Sollux blue filter lamp, irradiation distance 30-40 cm, duration 15 min; Bioptron lamp, irradiation distance 10 cm, duration 5-10 min

As mentioned before, the main therapeutic factor consisted in therapeutic radon- and sulfur-containing thermal waters which have been used in therapy for over 100 years. These are low-mineralized water with radon activity being the main therapeutic factor. Physicochemical characteristics of waters are presented in Table 1.

In Poland, health resort treatment is available only on physician's prescription and following a predefined dosage regimen after all indications and contraindications are taken into consideration. All procedures are identified in terms of their number, duration, and type. Radon health resorts make use of water originating from natural sources consisting in boreholes drilled in accordance with the Mining Law, upon approval of the Ministry of the Environment and under supervision of the Ministry of Health. In order to be considered as having therapeutic properties, each type of water must meet predefined balneochemical and bacteriological criteria.

As part of the study, changes in uric acid, bilirubin, and albumin levels were subject to observation as separate components of the antioxidative system. Standard assays were used in the analyses.

### 2.1. Albumin

Albumin and bromocresol green form a blue/green complex in acidic conditions. The intensity of the color is directly proportional to the concentration of albumin. The reagent consists of citrate buffer (pH 4.2), bromocresol green, inactive stabilizers, and detergents. Determinations are made on serum or plasma samples collected into EDTA-coated tubes. Albumin standard or a calibrator with values standardized for the bromocresol method is used as a control. Samples are placed in an automatic photometer that facilitates absorbance being measured at 630 (600-650) nm. After mixing the reagent and the plasma sample and incubating the resulting mixture for 2 minutes at 25°C, absorbance readouts are made using the reference sample (RS) and the test sample (TS). The albumin level is calculated from the following formula:

(1) $$\begin{array}{c} \text{Albumin level}=\frac{\text{A}\left(\text{TS}\right)}{\text{A}\left(\text{RS}\right)}\times \text{standard concentration}. \end{array}$$

Nowadays, determinations and calculations are performed automatically by computer systems. The methodology of the test procedure and calculation of albumin levels are included in the manual provided with the reagents.

### 2.2. Bilirubin

For the Jendrassik-Grof method, bilirubinreacts with a sulfanilic acid salt to form a dye which reacts with direct bilirubin present as water-soluble complexes and indirect albumin-bound bilirubin released by compounds that comprise the reagent. The intensity of coloration is proportional to the total concentration of bilirubin in the sample. The quality of determinations is compared to those of standard sera with total bilirubin levels standardized for the sulfanilic acid method. Composition of reagents:

*R1*: caffeine, sodium benzoate, and sodium acetate

*R2a*: sulfanilic acid, hydrochloric acid

*R2b*: sodium nitrite

Similar to the previously described determination, mixing the reagents is followed by the readout of photometric absorbance at 550 nm. Sequential addition of R1 and R2, each followed by incubation at 37°C for 2 minutes, leads to the following absorbance readouts: A1 (TS) and A2 (TS) for the test sample and A1 (RS) and A2 (RS) for the reference sample. The values are substituted to the formula

(2) $$\begin{array}{c} \text{Total bilirubin level}=\frac{\text{A}2\left(\text{TS}\right)-\text{A}1\left(\text{TS}\right)}{\text{A}2\left(\text{RS}\right)-\text{A}1\left(\text{RS}\right)}\times \text{standard concentration}. \end{array}$$

Nowadays, determinations and calculations are performed automatically by computer systems. The methodology of the test procedure and calculation of bilirubin levels are included in the manual provided with the reagents.

### 2.3. Uric Acid

The study material may consist of blood serum or plasma samples collected in heparin- or EDTA-coated tubes. Uricase is an enzyme catalyzing oxidation of uric acid to allantoin and hydrogen peroxide. Hydrogen peroxide reacts with TOS and 4-aminoantipyrine to form a colorful complex with color intensity being directly proportional to the level of uric acid. Composition of reagents:

*R1*: phosphate buffer (pH 7.6), EDTA, TOS, sodium azide

*R2*: phosphate buffer (pH 7.8), EDTA, uricase, peroxidase, 4-aminoantipyrine, sodium azide, inactive stabilizers and detergents

Reagents R1 and R2 should be mixed in a 4 : 1 ratio. The quality of determinations is verified by means of control sera with uric acid levels standardized for the uricase- and peroxidase-based enzymatic method. The obtained samples are placed an automatic photometer facilitating the measurement of absorbance at 530-570 nm. After mixing the reagent and the plasma sample and incubating the resulting mixture for 5 minutes at 37°C, absorbance readouts are made using the reference sample (RS) and the test sample (TS). Calculations:

(3) $$\begin{array}{c} \text{Uric acid level}=\frac{\text{A}\left(\text{TS}\right)}{\text{A}\left(\text{RS}\right)}\times \text{standard concentration}. \end{array}$$

The methodology of the test procedure and calculation of uric acid levels are included in the manual provided with the reagents.

The reference ranges of individual metabolites are as follows: albumin 3.5-5 g/dL, bilirubin 0.3-1.2 mg/dL, and uric acid 3.5-7 g/dL.

A control group was provided for in the study design. It consisted of a total of 15 subjects (10 females and 5 males) selected from among the resort employees, aged 24 to 58 years with the mean age of 41.7 years. Subjects included in the control group were healthy, did not take advantage of the resort treatment facilities, and did not smoke or drink alcohol. Main recommendation included continuation of previous lifestyle and a ban on taking advantage of the resort treatment facilities; otherwise, the same inclusion and exclusion criteria as well as the same diagnostic procedures applied.

The study was approved by the Bioethics Committee of the Wroclaw Medical University (opinion no. KB-401/2008) and received a written authorization from the President of the Uzdrowisko Przerzeczyn Sp.z o.o. health resort, and voluntary, individual, written consents from patients and control group subjects were pursuant to the consent sample recommended by the Bioethics Committee of the Wrocław Medical University. Study documentation is kept by the first author.

The results were processed by means of statistical analysis using a Polish-language version of STATISTICA software. The significance level was defined as *P* < 0.05. The sign test and Wilcoxon's signed-rank test were used, with tied pairs consisting of the “before treatment” and “after treatment” values obtained in the same patient. In cases of statistically significant differences, detailed information on the differences (between the “before treatment” and “after treatment” values) was provided using descriptive statistics, including the comparisons of means, medians, and the 25^th^ and 75^th^ percentiles.

## 3. Results

**Figure 1:**
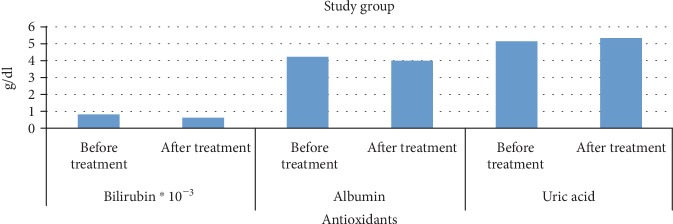
Changes in the mean plasma levels of bilirubin, uric acid, and albumin in study group patients.

Changes in all parameters were within the reference ranges. The mean plasma albumin level before the treatment in study group patients was 4.16 g/dL as compared to 4.02 g/dL after the treatment. The mean plasma bilirubin level before the treatment in study group patients was 0.81 mg/dL as compared to 0.61 mg/dL after the treatment. The mean plasma uric acid level before the treatment in study group patients was 5.05 g/dL as compared to 5.27 g/dL after the treatment (Figure 1).

**Figure 2:**
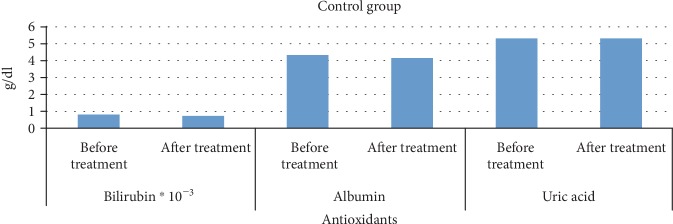
Changes in the mean plasma levels of bilirubin, uric acid, and albumin in control group subjects.

Mean plasma levels of albumin, bilirubin, and uric acid in the control group were as follows: 4.22 g/dL, 0.76 mg/dL, and 5.17 g/dL, respectively, before the treatment and 4.11 g/dL, 0.69 mg/dL, and 5.22 g/dL, respectively, after the treatment (Figure 2).

Wilcoxon's signed-rank test and sign test values obtained in the study group are presented in Tables 2 and 3. The results confirmed the statistical significance of changes in mean concentrations before and after the treatment.

Nonparametric tests and simple numerical comparisons were also used in the assessment of tested characteristics so as to determine which of the two values, i.e., the value determined before the treatment vs. the value determined after the treatment, was greater. This simple comparison turned out to be also reliable and appropriate as significant differences were observed between the “before treatment” and “after treatment” results for each statistical test. Medians, means, and the 25^th^ and 75^th^ percentiles were subsequently compared in the listed order. Values calculated to two decimal places are presented in Table 4.

Wilcoxon's signed-rank test and sign test values obtained in the control group are presented in Tables 5 and 6. The obtained results are not indicative of any statistically significant changes in the “before treatment” vs. the “after treatment” values of individual test parameters.

The descriptive statistics within the study groups illustrate the changes which could not have been demonstrated in nonparametric tests due to the lack of statistical significance (Table 7).

## 4. Discussion

Endogenous, low-molecular hydrophilic antioxidants, including uric acid and bilirubin, as well as plasma proteins, such as albumin, play an important role in protection against reactive oxygen species. Changes in the plasma levels of albumin, bilirubin, and uric acid were observed in the study, including a drop in the levels of albumin and bilirubin and an increase in the levels of uric acid. Uric acid is an important member of the class of low-molecular antioxidants. Hyperuricemia is indicative of oxidative stress, and uric acid is considered to be a strong antioxidant. Particularly highlighted is its neuroprotective effect consisting in restriction of accumulation of free radicals, maintenance of calcium equilibrium required in neural transmission, and stabilization of mitochondrial function; hence, appropriate treatments taking advantage of well-soluble uric acid analogs were attempted [32]. Studies in animal models confirmed the reduction in the incidence of brain damage following administration of uric acid analogs [33], while a contradictory potential of uric acid was suggested in in vivo studies. Some authors believe that increased levels of uric acid in elderly patients increase the risk and worsen the prognosis of brain stroke [34, 35], while other observations confirm clinical improvement in patients after ischemic brain stroke in whom an increase in uric acid levels was observed [36].

As demonstrated in studies conducted by Miller and Kedziora, plasma levels of uric acid and total systemic antioxidative potential increased following a series of 10 cryotherapy sessions in multiple sclerosis patients. The author believes that by increasing the total systemic antioxidative potential including the uric acid level, cryotherapy may constitute a supplementary treatment in MS patients [37]. This conclusion surely warrants further studies and observations. Uric acid plays an important role in prevention of oxidative stress during extensive physical effort. Antioxidative properties of uric acid were shown to be of biological importance in in vivo studies [38]. As shown by the studies conducted by Zielinski et al., uric acid, while being a metabolite of purin transformations in humans, is characterized by a slow, steady increase in plasma levels until long after completion of physical exercise [39].

As also shown in various studies, different factors may affect the plasma levels of uric acid thus pointing to its important role as a metabolite with antioxidative and cytoprotective properties. Antioxidative capacity of plasma and serum is 80% dependent on two types of compounds: one is uric acid which is responsible for 35-65% of the antioxidative capabilities of the system, while the other are proteins which are responsible to 10-50% of these capabilities. It is likely that the proteins and uric acid work together and complement one another in their antioxidative function. Albumin, being responsible for the maintenance of circulating blood volume and acting as a transportation system, plays an important role in this function. As mentioned before, albumin belongs to a group of proteins involved in antioxidative processes. It is characterized by good ROS scavenging efficacy, and its antioxidative properties are due to the presence of sulfhydryl groups. It is capable of binding metal ions and prevents formation of hydroxyl radicals from hydrogen peroxide [40, 41]. In our study, a reduction in the albumin levels was observed in the study group, although the values remained within the physiological reference range. This might be suggestive of albumin's involvement in free radical-mediated processes by means of binding the products of such reactions. Obviously, the reliability of this observation can be disputed since damaged albumin molecules are replaced by new molecules and the efficacy of systemic production of albumin may reach 3 grams per day. It is also known that drops in albumin levels may be due to catabolism, blood dilution, increased glomerular filtration, physical exercise, or synthesis being inhibited by toxins. Albumin is also a negative acute phase protein (i.e., a protein whose plasma levels decrease in response to inflammation). Besides inflammation, broadly understood stress is another factor capable of triggering changes in the acute phase protein levels.

Frih et al. demonstrated no changes in albumin levels following kinesitherapy in patients undergoing hemodialysis [42]. The study focused only on a single element of the overall treatment, i.e., kinesitherapy.

Bilirubin is not only the product of heme conversion but also an important, metabolically active compound. In our study, a decrease in bilirubin levels was observed in a manner similar to that observed for albumin. Products of hemoglobin transformations, particularly heme degradation products, are toxic at large concentrations. On the other hand, they were shown to exert cytoprotective effects at low levels. An example may be provided by bilirubin which, when present at low levels, is an anti-inflammatory and antioxidative agent acting via scavenging the reactive oxygen and nitrogen species in an electron transfer mechanism [43].

Not many studies could be found on the impact of various forms of balneotherapy on the nonenzymatic antioxidant system. It is assumed that the balneophysical stimuli are treated by the system as factors that trigger oxidative stress. This is followed by radical-scavenging reactions occurring at various levels of antioxidative protection, leading, e.g., to a reduction in albumin and bilirubin levels and to an increase in uric acid levels. Studies from other centers also confirmed the changes occurring within the antioxidative system following various forms of balneotherapy [44]. The exact mechanisms responsible for these phenomena could not be unambiguously explained to date. Few studies in the area of health resort medicine as well as other areas are suggestive of certain phenomena associated with free radical-mediated transformations. Stimulation of the antioxidant system was identified as an effect of balneotherapy following a query in scientific journal databases. In his studies conducted in the 1990s, Reinisch confirmed the impact of radon therapy on the antioxidant system. He observed a reduction in the release of ROS from neutrophilic leukocytes in patients with ankylosing spondylitis [45]. Yamaoka et al. demonstrated that small doses of radon stimulated the activity of certain enzymes comprising the antioxidative protection system, such as superoxide dismutase [46].

Therefore, a question can be raised as to whether oxidative stress caused by balneophysiotherapy is necessary and beneficial. As far as clinical evaluation is concerned, it surely is. The therapeutic effect is most likely due to the controlled disruption of systemic homeostasis as the result of comprehensive stimulation treatment delivered over a period of two weeks or more. The nature of the stimuli delivered as part of the health resort treatment is that of adaptive stress. This means that these lead to systemic adaptation or hardening. A possible beneficial effect may consist in an increase in the systemic immunity as the result of poorly understood mechanisms of transient functional or morphological changes that also depend on stimulation of the reticular activation system and the autonomic nervous system. This mechanism may provide a basis for positive systemic transformations that condition the improvement following health resort treatment [47]. One may expect that the increase in the immunity and physical fitness, the resolution of pain, normalization of blood pressure, and numerous other beneficial systemic effects are secondary to the impact of health resort balneophysiotherapy that stimulates the antioxidative system, with the results obtained in this study providing a signal of metabolic activity within the said system.

The levels of uric acid, albumin, and bilirubin are affected by numerous factors, and the measured values should not be interpreted in an unambiguous manner. These should not be the only elements of the antioxidant system taken into account as the primary indicator of free radical-involving transformations. With no doubt, the assessment of the overall and enzymatic antioxidative potentials of the system appears to be most reliable. However, changes observed in this study are indicative of metabolic changes activated by balneophysiotherapeutic stimuli.

## 5. Conclusions

1. Bilirubin and albumin levels were reduced while uric acid levels were increased as the result of health resort therapy in patients with degenerative motor organ diseases
2. The observed changes in the levels of nonenzymatic endogenous antioxidants depend on free radical-mediated systemic transformations triggered by balneophysiotherapy

## Figures and Tables

**Table 1:** Physicochemical assessments of therapeutic waters as sampled at water springs and within the natural therapy center (NTC).

No.	Sampling site	Water temperature (°C)	pH	Content per 1 dm^3^ of water			
				H_2_S Mg	HCO_3_ Mg	Rn NCi	Rn Bq
1	Borehole II	12.0	7.62	1.96	263.2	2.21	81.8
2	Borehole IX	12.0	7.72	1.70	289.6	1.71	63.3
3	NTC bath	16.0	7.65	1.87	277.9	2.20	81.4

**Table 2:** Wilcoxon's test—evaluation of changes in mean albumin, bilirubin, and uric acid levels in the study group.

	Albumin	Bilirubin	Uric acid
*Z*	-6.844^b^	-6.714^b^	-3.285^a^
Asymptotic significance (2-sided) *P*	<0.001	<0.001	0.001
Exact significance (2-sided) *P*	<0.001	<0.001	0.001

**Table 3:** Sign test—evaluation of changes in mean albumin, bilirubin, and uric acid levels in the study group.

	Albumin	Bilirubin	Uric acid
*Z*	-6.834	-6.264	-3.628
Asymptotic significance (2-sided) *P*	<0.001	<0.001	<0.001
Exact significance (2-sided) *P*	<0.001	<0.001	<0.001

**Table 4:** Descriptive statistics—“before treatment” and “after treatment” values of plasma albumin, bilirubin, and uric acid levels in study group patients.

	*N*	Mean	Standard deviation	Minimum	Maximum	Percentile		
						25^th^	50^th^	75^th^
Albumin before	110	4.16	0.21	3.40	4.60	4.00	4.20	4.30
Bilirubin before	110	0.81	0.39	0.31	2.88	0.56	0.68	0.93
Uric acid before	110	5.05	1.08	3.00	8.80	4.20	5.00	5.70
Albumin after	110	4.02	0.30	3.40	6.30	3.90	4.00	4.20
Bilirubin after	110	0.61	0.26	0.25	1.71	0.44	0.55	0.75
Uric acid after	110	5.27	1.18	2.70	9.50	4.50	5.00	6.10

**Table 5:** Wilcoxon's test—evaluation of changes in mean albumin, bilirubin, and uric acid levels in the control group.

	Albumin	Bilirubin	Uric acid
*Z*	-1.912^a^	-0.691^a^	-0.432^b^
Exact significance (2-sided) *P*	0.065	0.511	0.691

**Table 6:** Sign test—evaluation of changes in mean albumin, bilirubin, and uric acid levels in the control group.

	Albumin	Bilirubin	Uric acid
Exact significance (2-sided) *P*	0.146	1	1

**Table 7:** Descriptive statistics—“before treatment” and “after treatment” values of plasma albumin, bilirubin, and uric acid levels in the control group subjects.

	*N*	Mean	Standard deviation	Minimum	Maximum	Percentile		
						25^th^	50^th^	75^th^
Albumin before	15	4.22	0.27	3.60	4.80	4.15	4.25	4.40
Albumin after	15	4.11	0.20	3.80	4.50	3.97	4.10	4.30
Bilirubin before	15	0.76	0.48	0.37	2.22	0.39	0.68	0.90
Bilirubin after	15	0.69	0.35	0.34	1.51	0.45	0.55	0.83
Uric acid before	15	5.17	1.45	3.00	7.80	4.10	5.15	6.40
Uric acid after	15	5.22	1.25	3.40	7.50	4.10	5.25	6.27

## Data Availability

All data are contained and described within the manuscript. The datasets used and/or analyzed during the current study are available from the corresponding author on reasonable request.

## Additional Points

*Trial Registration*. The number of clinical trial is NCT03405350. All information about this study is available under this number. The study was carried out as part of the statutory task SUB.E060.19.001.

## Supplementary Materials

Supplementary Materials Results of measurements of albumin, bilirubin, and uric acid during the examination in SPA Przerzeczyn Zdrój.
