# Supplementary material for: The Impact of Health Resort Treatment on the Nonenzymatic Endogenous Antioxidant System
Source: Oxid Med Cell Longev. 2020 Jan 31;2020:8423105. doi: 10.1155/2020/8423105 (PMC7016389; doi:10.1155/2020/8423105)
Supplement: Supplementary Materials — Results of measurements of albumin, bilirubin, and uric acid during the examination in SPA Przerzeczyn Zdrój. [file 8423105.f1.doc]

WYNIKI do obliczeń– KWAS MOCZOWY GRUPA BADANA I GRUPA KONTROLNA

Przerzeczyn -Zdrój

|  |  | **Kwas moczowy** | | | **mg/dl 3,5-7,2** |
| --- | --- | --- | --- | --- | --- |
| **Nr pacjenta** | **płeć** | **Badanie przed terapią** |  | | Badanie po terapii |
| **1** | **K** | **3,5** |  | | **4,5** |
| **2** | **M** | **5,5** |  | | 6,5 |
| **3** | **K** | **6,9** |  | | 7,2 |
| **4** | **M** | **4,2** |  | | Brak osocza |
| **5** | **K** | **4,5** |  | | 5 |
| **6** | **K** | **4,4** |  | | 4,8 |
| **7** | **K** | **5,1** |  | | 5,3 |
| **8** | **K** | **5,0** |  | | 5,5 |
| **9** | **K** | **5,3** |  | | 4,8 |
| **10** | **K** | **3,8** |  | | 4,9 |
| **11** | **k** | **Brak osocza** |  | |  |
| **12** | **m** | **Brak osocza** |  | |  |
| **13** | **M** | **6,6** |  | | 6,9 |
| **14** | **m** | **Brak osocza** |  | |  |
| **15** | **K** | **5,4** |  | | 5,8 |
| **16** | **K** | **3,8** |  | | 3,8 |
| **17** | **K** | **4,3** |  | | 4,5 |
| **18** | **K** | **4,1** |  | | 4,7 |
| **19** | **M** | **5,1** |  | | 6,2 |
| **20** | **k** | **Brak osocza** |  | |  |
| **21** | **K** | **5,5** |  | | 4,6 |
| **22** | **K** | **3,9** |  | | 4 |
| **23** | **K** | **Kontrola 6,7** |  | | 6,2 |
| **24** | **K** | **5,2** |  | | 4,8 |
| **25** | **K** | **5,7** |  | | 4,9 |
| **26** | **K** | **3,9** |  | | 4,2 |
| **27** | **K** | **6,2** |  | | 6,9 |
| **28** | **K** | **4,1** |  | | 3,6 |
| **29** | **K** | **7,3** |  | | 6,3 |
| **30** | **M** | **Brak osocza** |  | |  |
| **31** | **k** | **Brak osocza** |  | |  |
| **32** | **K** | **4,2** |  | | 4,5 |
| **32x** | **K** | **4,5** |  | | 4,1 |
| **33** | **K** | **3,9** |  | | 4,2 |
| **34** | **K** | **6,3** |  | | 5,9 |
| **35** | **K** | **3,2** |  | | 4,5 |
| **36** | **K** | **Kontrola 4,3** |  | | 4,3 |
| **37** | **K** | **Kontrola 4,4** |  | | 4,2 |
| **38** | **M** | **Kontrola 7,8** |  | | 6,5 |
| **39** | **K** | **Kontrola 3,0** |  | | 3,4 |
| **40** | **M** | **4,2** |  | | 4,4 |
| **41** | **M** | **Kontrola 6,8** |  | | 7,5 |
| **42** | **K** | **4,5** |  | | 4,8 |
| **43** | **M** | **5,8** |  | | 5,2 |
| **44** | **K** | **3,8** |  | | 3,8 |
| **45** | M | Kontrola 5,4 |  | 6,7 | |
| **46** | **M** | **4,4** |  | | Brak osocza |
| **47** | **K** | **5,7** |  | | 5 |
| **48** | **M** | **8,2** |  | | 6,2 |
| **49** | **k** | **Kontrola 4,7** |  | | 5,5 |
| **50** | **K** | **4,0** |  | | 5,5 |
| **51** | **K** | **4,3** |  | | 2,7 |
| **52** | **M** | **Brak osocza** |  | |  |
| **53** | **M** | **4,6** |  | | 5 |
| **54** | **K** | **4,7** |  | | 4 |
| **55** | **K** | **6,4** |  | | 6,1 |
| **56** | **M** | **5,9** |  | | 6,1 |
| **57** | **K** | **5,6** |  | | 7 |
| **58** | **M** | **Kontrola 6,3** |  | | 6,0 |
| **59** | **K** | **Kontrola 3,5** |  | | 3,8 |
| **60** | **K** | **Kontrola 5,6** |  | | 5,1 |
| **61** | **K** | **5,2** |  | | 4,9 |
| **62** | **K** | **Kontrola 6,0** |  | | 5,4 |
| **63** | **K** | **3,8** |  | | 3,7 |
| **64** | **K** | 4,0 |  | | 4,1 |
| **65** | **K** | **5,3** |  | | 5,5 |
| **66** | **K** | **4,4** |  | | 4 |
| **67** | **K** | **5,6** |  | | 8,3 |
| **68** | **M** | **6,7** |  | | 7 |
| **69** | **M** | **5,0** |  | | 5,9 |
| **70** | **K** | **3,6** |  | | 3,8 |
| **71** | **M** | **5,2** |  | | 6,4 |
| **72** | **K** | **3,1** |  | | 4 |
| **73** | **K** | **Brak osocza** |  | |  |
| **74** | **K** | **6,1** |  | | 6,1 |
| **75** | **M** | **5,4** |  | | 4,5 |
| **76** | **K** | **5,0** |  | | 4,8 |
| **77** | **K** | **5,1** |  | | 5,3 |
| **78** | **K** | **5,5** |  | | 6,6 |
| **79** | **K** | **3,5** |  | | 4 |
| **80** | **K** | **4,2** |  | | 4,7 |
| **81** | **K** | **4,2** |  | | 4,7 |
| **82** | **M** | **5,7** |  | | 7,4 |
| **83** | **M** | **6,3** |  | | 6,5 |
| **84** | **K** | **4,1** |  | | 4,7 |
| **85** | **M** | **5,5** |  | | 5,4 |
| **86** | **K** | **5,2** |  | | 4,9 |
| **87** | **K** | **4,6** |  | | 4,4 |
| **88** | **M** | **4,1** |  | | 5,1 |
| **89** | **M** | **6,3** |  | | 8,9 |
| **90** | **K** | **4,9** |  | | 5 |
| **91** | **M** | **5,0** |  | | 4,9 |
| **92** | **M** | **3,2** |  | | 3,6 |
| **93** | **M** | **6,1** |  | | 7 |
| **94** | **K** | **5,0** |  | | 5,4 |
| **95** | **M** | **6,6** |  | | 5,8 |
| **96** | **K** | **6,3** |  | | 6,4 |
| **97** | **M** | **4,0** |  | | 4,5 |
| **98** | **M** | **5,2** |  | | 5,2 |
| **99** | **K** | **3,9** |  | | 4,3 |
| **100** | **M** | **6,8** |  | | 7 |
| **101** | **K** | **5,3** |  | | 4,7 |
| **102** | **K** | **Kontrola 4,9** |  | | 4,9 |
| **103** | **K** | **5,2** |  | | 5,5 |
| **104** | **K** | **3,7** |  | | 4,1 |
| **105** | **K** | **3,9** |  | | 4,4 |
| **106** | **M** | **7,2** |  | | 6,7 |
| **107** | **K** | **5,4** |  | | 5 |
| **108** | **M** | **4,8** |  | | 5,4 |
| **109** | **K** | **5,7** |  | | 5,8 |
| **110** | **M** | **6,5** |  | | 6,4 |
| **111** | **K** | **4,3** |  | | 4,5 |
| **112** | **K** | **4,4** |  | | 4,9 |
| **113** | **M** | **3,8** |  | | 4,5 |
| **114** | **K** | **4,8** |  | | 4,5 |
| **115** | **K** | **4,8** |  | | 4,8 |
| **116** | **M** | **6,9** |  | | 8,3 |
| **117** | **K** | **5,3** |  | | 5,1 |
| **118** | **K** | **4,8** |  | | 5 |
| **119** | **M** | **Kontrola 4,5** |  | | 5,4 |
| **120** | **K** | **4,0** |  | | 4,5 |
| **121** | **K** | **5,5** |  | | 6,3 |
| **122** | **K** | 3,1 |  | | 3,7 |
| **123** | **K** | **3,9** |  | | 4,1 |
| **124** | **K** | **4,8** |  | | 4,2 |
| **125** | **K** | **5,6** |  | | 5,8 |
| **126** | **M** | **4,6** |  | | 4,7 |
| **127** | **K** | **4,4** |  | | 4,4 |
| **128** | **K** | **4,1** |  | | 4,3 |
| **129** | **M** | **5,6** |  | | 6 |
| **130** | **K** | **4,9** |  | | 3,9 |
| **131** | **M** | **6,4** |  | | 6,6 |
| **132** | **K** | **3,0** |  | | 4,4 |
| **133** | **K** | **6,9** |  | | 6,8 |
| **134** | **M** | **8,8** |  | | 9,5 |
| **135** | **M** | **Brak osocza** |  | |  |
| **136** | **K** | **5,8** |  | | 5,8 |
| **137** | **K** | Brak osocza |  | |  |
| **138** | **K** | Brak osocza |  | |  |

WYNIKI –do obliczeń-ALBUMINA I BILIRUBINA GRUPA BADANA I GRUPA KONTROLNA

Przerzeczyn -Zdrój

| Numer pacjenta |  | **ALBUMINA**  **G/DL 3,5-5,2** | | | |  | Bilirubina  mg/dl 0,1-1,3 | | |
| --- | --- | --- | --- | --- | --- | --- | --- | --- | --- |
|  |  |  | | | |  |  | | |
|  | **płeć** |  | | **Przed** | **po** |  | **przed** | **po** |  |
| **1** | **K** |  | | **4** | **3,9** |  | 0,52 | **0,39** |  |
| **2** | **M** |  | | **4,2** | **3,9** |  | **0,62** | 0,5 |  |
| **3** | **K** |  | | **4,0** | **4,0** |  | **0,49** | 0,54 |  |
| **4** | **M** |  | | **4,2** | **brak osocza** | | 0,71 | brak osocza | |
| **5** | **K** |  | | **3,9** | **4,0** |  | 0,62 | 0,72 |  |
| **6** | **K** |  | | **4,2** | **4,1** |  | 0,66 | 0,65 |  |
| **7** | **K** |  | | **4,2** | **4,3** |  | 0,86 | 0,58 |  |
| **8** | **K** |  | | **3,8** | **3,7** |  | 0,55 | 0,87 |  |
| **9** | **K** |  | | **4** | **4** |  | 0,74 | 0,33 |  |
| **10** | **K** |  | | **4** | **3,6** |  | 0,64 | 0,26 |  |
| **11** | **K** |  | | **3,9** | **brak osocza** | | 0,39 | Brak osocza | |
| **12** | **M** |  | | **4,1** | **brak osocza** | | 0,64 | brak osocza | |
| **13** | **M** |  | | **4,3** | **4,4** |  | 1,88 | 0,64 |  |
| **14** | **M** |  | | **4,2** | **brak osocza** | | 1,21 | Brak osocza | |
| **15** | **K** |  | | **3,8** | **3,7** |  | 0,86 | 0,73 |  |
| **16** | **K** |  | | **4,6** | **4,1** |  | 0,48 | 0,36 |  |
| **17** | **K** |  | | **4** | **3,8** |  | 0,52 | 0,49 |  |
| **18** | **K** |  | | **4,1** | **4,3** |  | 0,69 | 0,53 |  |
| **19** | **M** |  | | **4,2** | **4,3** |  | 0,63 | 0,44 |  |
| **20** | **K** |  | | **3,7** | **brak osocza** | | 0,52 | brak osocza | |
| **21** | **K** |  | | **3,9** | **3,7** |  | 0,68 | 0,57 |  |
| **22** | **K** |  | | **4,4** | **4,2** |  | 0,68 | 0,65 |  |
| **23** | **K** | **Kontrola** | | **3,9** | **3,8** | **Kontrola** | 0,71 | 0,46 |  |
| **24** | **K** |  | | **4,6** | **4,3** |  | 0,39 | 0,5 |  |
| **25** | **K** |  | | **4,2** | **4,1** |  | 0,4 | 0,6 |  |
| **26** | **K** |  | | **4,1** | **4,1** |  | 0,49 | 0,53 |  |
| **27** | **K** |  | | **3,4** | **3,4** |  | 0,4 | 0,53 |  |
| **28** | **K** |  | | **4,4** | **4,3** |  | 1,25 | 0,59 |  |
| **29** | **K** |  | | **4,2** | **4** |  | 1,16 | 0,44 |  |
| **30** | **M** |  | | **4,3** | **brak osocza** | | 0,45 | Brak osocza | |
| **31** | **K** |  | | **4,1** | **brak osocza** | | 0,43 | brak osocza | |
| **32** | **K** |  | | **4,3** | **4,4** |  | **0,59** | 0,38 |  |
| **32x** | **K** |  | | **4,5** | **4,7** |  | **0,59** | 0,86 |  |
| **33** | **K** |  | | **3,9** | **4,2** |  | 0,53 | 0,45 |  |
| **34** | **K** |  | | **4,3** | **4** |  | 0,55 | 0,43 |  |
| **35** | **K** |  | | **4,6** | **4,5** |  | 0,63 | 0,49 |  |
| **36** | **K** | **Kontrola** | | **4,2** | **4** | **Kontrola** | 1,1 | **0,81** |  |
| **37** | **K** | **Kontrola** | **4,2** | | **4,1** | **Kontrola** | 0,37 | 0,38 |  |
| **38** | **M** | **Kontrola** | **4,2** | | **3,9** | **Kontrola** | 0,86 | 0,59 |  |
| **39** | **K** | **Kontrola** | **4,3** | | **4,1** | **Kontrola** | 0,63 | 0,34 |  |
| **40** | **M** |  | | **3,9** | **3,9** |  | 1,01 | 1,04 |  |
| **41** | **M** | **Kontrola** | **4,4** | | **4,5** | **Kontrola** | 0,77 | 1,32 |  |
| **42** | **K** |  | | **4** | **3,9** |  | 0,77 | 0,55 |  |
| **43** | **M** |  | | **4,4** | **4,2** |  | 1,51 | 0,73 |  |
| **44** | **K** |  | | **4,6** | **4,4** |  | 0,31 | 0,27 |  |
| **45** | M | **Kontrola** | | **4,8** | 4,3 | Kontrola | **0,78** | 0,74 |  |
| **46** | **M** |  | | **brak** |  | **osocza** |  |  |  |
| **47** | **K** |  | | **4,1** | **4,3** |  | 0,53 | 0,44 |  |
| **48** | **M** |  | | **4,4** | **4,3** |  | 0,67 | 0,5 |  |
| **49** | **K** | **Kontrola** | **4,4** | | **4,1** | **Kontrola** | 2,22 | 1,51 |  |
| **50** | **K** |  | | **4,2** | **4,2** |  | 0,64 | 0,61 |  |
| **51** | **K** |  | | **4,4** | **4,2** |  | 0,56 | 0,54 |  |
| **52** | **m** |  | | **brak** |  | **osocza** |  |  |  |
| **53** | **M** |  | | **4,2** | **3,9** |  | 1,5 | 1,05 |  |
| **54** | **K** |  | | **4** | **3,9** |  | 1,49 | 0,85 |  |
| **55** | **K** |  | | **4,3** | **4** |  | 1,4 | 1,14 |  |
| **56** | **M** |  | | **4,3** | **4,2** |  | 0,82 | 0,77 |  |
| **57** | **K** |  | | **4,6** | **4,3** |  | 0,56 | 0,67 |  |
| **58** | **M** | **Kontrola** | | **4,2** | **4,0** | **Kontrola** | 1,02 | 0,43 |  |
| **59** | **K** | **Kontrola** | | **4,0** | **4,2** | **Kontrola** | 0,4 | 0,5 |  |
| **60** | K | **Kontrola** | | **3,6** | **3,8** | **Kontrola** | 0,38 | 0,51 |  |
| **61** | **K** |  | | **4** | **3,8** |  | 0,48 | 0,53 |  |
| **62** | **K** | **Kontrola** | | **4,3** | **4,3** | **Kontrola** | 0,39 | 0,46 |  |
| **63** | **K** |  | | **4,1** | **4,0** |  | 0,72 | 0,36 |  |
| **64** | **K** |  | | **3,9** | **4,0** |  | 0,6 | 0,52 |  |
| **65** | **K** |  | | **3,9** | **3,9** |  | 0,86 | 0,72 |  |
| **66** | **K** |  | | **4,6** | **4,2** |  | 0,98 | 0,62 |  |
| **67** | **K** |  | | **4,1** | **4,2** |  | 0,54 | 0,68 |  |
| **68** | **M** |  | | **4,2** | **3,8** |  | 1,48 | 0,72 |  |
| **69** | **M** |  | | **4,1** | **3,8** |  | 2,88 | 1,17 |  |
| **70** | **K** |  | | **4,2** | **4,2** |  | 0,65 | 0,87 |  |
| **71** | **M** |  | | **4,2** | **3,9** |  | 1,35 | 1,53 |  |
| **72** | **K** |  | | **4,2** | **4** |  | 0,69 | 0,29 |  |
| **73** | **K** |  | brak | |  | osocza |  |  |  |
| **74** | **K** |  | | **4,1** | **3,7** |  | 0,64 | 0,34 |  |
| **75** | **M** |  | | **4,4** | **4,1** |  | 0,73 | 0,43 |  |
| **76** | **K** |  | | **4,2** | **4,0** |  | 0,57 | 0,48 |  |
| **77** | **K** |  | | **3,9** | **3,8** |  | 0,54 | 0,44 |  |
| **78** | **K** |  | | **4,1** | **3,8** |  | 0,86 | 0,5 |  |
| **79** | **K** |  | | **4,1** | **3,9** |  | 0,89 | 0,79 |  |
| **80** | **K** |  | | **3,8** | **3,8** |  | 0,86 | 0,76 |  |
| **81** | **K** |  | | **4,1** | **3,9** |  | 0,32 | 0,42 |  |
| **82** | **M** |  | | **4,2** | **3,9** |  | 0,58 | 0,42 |  |
| **83** | **M** |  | | **4,4** | **4,0** |  | 0,77 | 0,56 |  |
| **84** | **K** |  | | **4,2** | **3,9** |  | 1,22 | 1,01 |  |
| **85** | **M** |  | | **4,4** | **4,3** |  | 1,62 | 1,71 |  |
| **86** | **K** |  | | **4,1** | **4,0** |  | 1,15 | 1,03 |  |
| **87** | **K** |  | | **4,5** | **3,9** |  | 1,35 | 0,51 |  |
| **88** | **M** |  | | **4,4** | **4,1** |  | 0,81 | 0,57 |  |
| **89** | **M** |  | | **4,1** | **6,3** |  | 1,93 | 0,84 |  |
| **90** | **K** |  | | **4,0** | **4,0** |  | 0,71 | 0,56 |  |
| **91** | **M** |  | | **4,5** | **4,0** |  | 0,85 | 0,83 |  |
| **92** | **M** |  | | **4,2** | **4,0** |  | 0,58 | 0,46 |  |
| **93** | **M** |  | | **3,9** | **3,8** |  | 0,93 | 0,6 |  |
| **94** | **K** |  | | **4,3** | **3,9** |  | 0,67 | 0,43 |  |
| **95** | **M** |  | | **4,6** | **4,1** |  | 1,02 | 0,76 |  |
| **96** | **K** |  | | **3,9** | **3,6** |  | 0,75 | 0,53 |  |
| **97** | **M** |  | | **4,3** | **3,8** |  | 1,1 | 0,82 |  |
| **98** | **M** |  | | **4,3** | **4,2** |  | 0,66 | 0,8 |  |
| **99** | **K** |  | | **4,0** | **3,9** |  | 0,82 | 0,63 |  |
| **100** | **M** |  | | **4,2** | **4,3** |  | 0,8 | 0,63 |  |
| **101** | **K** |  | | **4,4** | **4,4** |  | 0,74 | 0,36 |  |
| **102** | **K** | Kontrola | | 4,3 | 4,3 | **Kontrola** | 0,66 | 0,7 |  |
| **103** | **K** |  | | **3,9** | **3,7** |  | 0,56 | 0,4 |  |
| **104** | **K** |  | | **4,1** | **4,0** |  | 0,67 | 0,35 |  |
| **105** | **K** |  | | **3,8** | **3,5** |  | 0,67 | 0,63 |  |
| **106** | **M** |  | | **4,3** | **4,0** |  | 1,18 | 0,75 |  |
| **107** | **K** |  | | **4,3** | **4,2** |  | 0,42 | 0,43 |  |
| **108** | **M** |  | | **4,1** | **4,0** |  | 0,68 | 0,55 |  |
| **109** | **K** |  | | **4,2** | **3,9** |  | 0,94 | 0,8 |  |
| **110** | **M** |  | | **4,3** | **4,0** |  | 1,02 | 0,36 |  |
| **111** | **K** |  | | **4,2** | **4,1** |  | 0,56 | 0,33 |  |
| **112** | **K** |  | | **4,1** | **3,9** |  | 0,66 | 0,54 |  |
| **113** | **M** |  | | **4,2** | **4,3** |  | 0,95 | 0,77 |  |
| **114** | **K** |  | | **4,3** | **4,2** |  | 0,72 | 0,5 |  |
| **115** | **K** |  | | **4,0** | **3,8** |  | 0,66 | 0,36 |  |
| **116** | **M** |  | | **4,0** | **3,9** |  | 0,81 | 0,52 |  |
| **117** | **K** |  | | **4,0** | **4,1** |  | 0,97 | 0,79 |  |
| **118** | **K** |  | | **4,1** | **4,0** |  | 0,53 | 0,51 |  |
| **119** | **M** | **Kontrola** | | **4,1** | **4,1** | Kontrola | 0,5 | 0,55 |  |
| **120** | **K** |  | | **4,0** | **3,9** |  | 0,86 | 0,5 |  |
| **121** | **K** |  | | **4,3** | **3,8** |  | 0,56 | 0,25 |  |
| **122** | **K** | **Kontrola** | | 4,4 | 4,2 | Kontrola | 0,41 | 0,91 |  |
| **123** | **K** |  | | **4,2** | **3,9** |  | 0,73 | 0,33 |  |
| **124** | **K** |  | | **4** | **3,7** |  | 1,11 | 0,76 |  |
| **125** | **K** |  | | **4,4** | **4,1** |  | 0,89 | 0,41 |  |
| **126** | **M** |  | | **4,4** | **4,3** |  | 1,89 | 1,52 |  |
| **127** | **K** |  | | **4,4** | **3,9** |  | 0,34 | 0,26 |  |
| **128** | **K** |  | | **4,2** | **4,1** |  | 0,33 | 0,55 |  |
| **129** | **M** |  | | **4,4** | **4,1** |  | 0,61 | 0,54 |  |
| **130** | **K** |  | | **3,8** | **3,7** |  | 0,6 | 0,56 |  |
| **131** | **M** |  | | **4,2** | **3,9** |  | 0,49 | 0,33 |  |
| **132** | **K** |  | | **3,9** | **3,8** |  | 0,49 | 0,52 |  |
| **133** | **K** |  | | **3,8** | **3,7** |  | 1,41 | 1,11 |  |
| **134** | **M** |  | | **4,0** | **3,9** |  | 0,88 | 1,07 |  |
| **135** | **m** |  | | **brak** |  | osocza |  |  |  |
| **136** | **K** |  | | **4,3** | **4,1** |  | 0,6 | 0,31 |  |
| **137** | **K** |  | | **brak** |  | osocza |  |  |  |
| **138** | **K** |  | | **brak** |  | Osocza |  |  |  |
